# The Associations of Trunk Intramuscular Adipose Tissue Content with Dietary Intake and Eating Behavior in Younger and Older Japanese Women: A Pilot Study

**DOI:** 10.3390/nu18121867

**Published:** 2026-06-10

**Authors:** Funa Kitagawa, Erika Sando, Teruhiko Koike, Hiroshi Akima, Noriko Tanaka

**Affiliations:** 1 Graduate School of Education and Human Development, Nagoya University, Furo-cho, Chikusa, Nagoya 464-8601, Aichi, Japan; 2 Japan Society for the Promotion of Science (JSPS), Kojimachi, Chiyoda 102-0083, Tokyo, Japan; 3 Research Center of Health, Physical Fitness and Sports, Nagoya University, Furo-cho, Chikusa, Nagoya 464-8601, Aichi, Japan; 4 Graduate School of Medicine, Nagoya University, Furo-cho, Chikusa, Nagoya 464-8601, Aichi, Japan

**Keywords:** ectopic fat, skeletal muscle quality, dietary habits, magnetic resonance imaging, protein, aging

## Abstract

**Background/Objectives**: Intramuscular adipose tissue (IntraMAT) is the ectopic fat which accumulates within skeletal muscle. The relationship between trunk IntraMAT content and dietary intake was shown to differ with age in men, but it remains unclear the relationship in women. Therefore, the present study investigated the associations of IntraMAT content with dietary intake and eating behavior in younger and older women. **Methods**: This cross-sectional study involved 24 young women aged 18 to 23 years (body mass index (BMI): 20.5 ± 2.3 kg/m^2^) and 25 older women aged 66 to 77 years (BMI: 21.7 ± 2.5 kg/m^2^) who participated. IntraMAT content was assessed by magnetic resonance imaging at the height of the 3rd lumbar vertebra. Dietary intake was evaluated using a self-administered diet history questionnaire. Eating behavior was evaluated by scores calculated using the eating behavior questionnaire in the guideline for obesity (2022). Blood properties related to metabolic syndrome were also measured. **Results**: In the younger group, IntraMAT content was significantly related to HDL cholesterol and insulin (r_s_ = −0.411 and 0.415, *p* < 0.05). In the older group, IntraMAT content significantly correlated with the percentage of energy from protein, sense of hunger, and total eating behavior (r_s_ = −0.410 to 0.412, *p* < 0.05). **Conclusions**: Trunk IntraMAT content may be related to dietary protein intake and eating behavior in the older group.

## 1. Introduction

Dietary habits, including dietary intake and eating behavior, generally affect adipose tissue content [1,2]. Previous studies reported that intramuscular adipose tissue (IntraMAT) content correlated with dietary intake, such as protein [3] and saturated fatty acids [4]. The relationship between trunk IntraMAT content and dietary intake was shown to differ with age in men [5] and between the sexes in younger individuals [4]. However, it remains unclear whether age-related differences exist in the relationship between trunk IntraMAT content and dietary intake in healthy women. Post-prandial muscle protein synthesis rates were found to be significantly lower in older individuals than in younger individuals [6]. Aging is associated with a reduction in the muscle anabolic capacity, which is an important physiological mechanism underlying sarcopenia [7]. The effects of dietary intake on mortality and physical function were found to differ depending on age [8]. It seems very clear that there is a difference in IntraMAT content between older and younger individuals. Taken together, these findings imply that the relationship between dietary intake and IntraMAT content is unlikely to be consistent across populations of different ages. Therefore, it is imperative to examine these associations within younger and older groups separately, rather than relying solely on direct comparisons between them, to more accurately characterize age-specific patterns.

Eating behavior is one of the daily habits related to adipose tissue content. It is a broad term that encompasses food choices and motives, such as recognition of the body composition, motivation for eating, substitute eating, and feeling of fullness [9,10]. Previous studies reported that individuals with worse eating behaviors had a higher visceral adipose tissue cross-sectional area (CSA) and were at a higher risk of developing lifestyle-related diseases, such as metabolic syndrome [1], hypertension, obesity, and type 2 diabetes [10] than those with better eating behaviors. On the other hand, IntraMAT CSA correlated with visceral adipose tissue CSA [11] and the prevalence of insulin resistance [12]. In addition, not only visceral adipose tissue content, but also IntraMAT content correlated with metabolic risk factors, such as glucose, insulin, triglycerides, total cholesterol, and high-density lipoprotein (HDL) cholesterol [13,14,15]. Therefore, similar to visceral adipose tissue, IntraMAT content may be associated with eating behavior. However, the relationship between IntraMAT content and eating behavior remains unclear. Furthermore, studies on the relationship between IntraMAT content and eating behavior are warranted from the perspective of preventing cardiometabolic diseases.

The present study investigated the associations of IntraMAT content with dietary intake and eating behavior in younger and older Japanese women. The present study is a pilot study aimed at deriving preliminary hypotheses regarding these relationships. Previous studies demonstrated that metabolic risk factors significantly correlated with IntraMAT content [5,16]. Therefore, these factors need to be included in studies on IntraMAT content. Kitagawa et al. (2022) found that dietary intake correlated with IntraMAT content in older men [4], but not in younger men. Therefore, we hypothesized that dietary intake and eating behavior may correlate with IntraMAT content, but only in older women.

## 2. Materials and Methods

### 2.1. Participants

Forty-nine healthy Japanese women aged 18 to 77 years with no history of cerebral or cardiac diseases and the ability to walk independently without assistance were enrolled. Participants were classified into two groups: a younger group (*n* = 24, 19.9 ± 1.5 years) and older group (*n* = 25, 72.4 ± 3.2 years). Each participant visited the laboratory twice, once for magnetic resonance (MR) imaging and the other for residual measurements, within 7 days. The order of measurements for each participant was randomized. The present study was approved by the Ethics Committee of the Research Center of Health, Physical Fitness and Sports, our University (23–04) and was performed in accordance with the principles of the Declaration of Helsinki. Prior to data collection, we calculated the minimum sample size required for correlations, an effect size of 0.5, a significance level of 0.05, and a power level of 0.8. As a result, the minimum number for the sample size was estimated as 16 for each group.

### 2.2. Morphological Measurements

In addition to height, body weight and body fat percentage were measured by InBody (ITO-InBody 370, Ito Co., Ltd., Tokyo, Japan). Waist circumference at the height of the umbilicus was measured in the standing position with a non-stretchable tape.

### 2.3. MR Image Acquisition

A 3.0 Tesla whole-body MR image scanner (MAGNETOM Verio, Siemens Healthcare Diagnostics K.K., Tokyo, Japan) was used to take images of the trunk at the height of the third lumbar vertebra. The two-point Dixon method was performed using MR images acquired with the following parameters: repetition time: 4.5 ms, echo time: 1.225 and 2.450 ms, flip angle: 9°, matrix size: 255 × 255 mm, field of view: 320 × 512 mm, slice thickness: 5 mm. Participants were in the supine position and held their breath for approximately 15 s after an inspiration to reduce artifacts caused by respiratory motion during scans.

MR images were analyzed using SliceOmatic 5.0 software (TomoVision, Magog, QC, Canada). The CSAs of skeletal muscle tissue in 4 skeletal muscle groups, i.e., the rectus abdominis, abdominal oblique, erector spinae, and iliopsoas, were calculated by the region growing, 2D, and morphology functions of SliceOmatic [16]. IntraMAT content in each skeletal muscle group was calculated using the following equation [17]:

IntraMAT content (%) = [(Fat mean intensity)/(Water mean intensity) + (Fat mean intensity)] × 100

We calculated average IntraMAT content in the 4 skeletal muscle groups and used them in subsequent analyses. Test–retest reliabilities (ICC1,1) was 0.971 for IntraMAT content (*n* = 10).

### 2.4. Dietary Intake and Eating Behavior

Dietary intake in the previous month was assessed using the brief-type self-administered diet history questionnaire (BDHQ) for Japanese individuals. Details on the structure and calculation method of dietary intake by BDHQ were described elsewhere [18,19]. We focused on total energy intake and the percentages of energy from protein, animal protein, plant protein, fat, animal fat, plant fat, saturated fatty acids, monounsaturated fatty acids, polyunsaturated fatty acids, and carbohydrates. In the older group, cognitive function was screened prior to enrollment, and participants who did not meet the predefined cutoff were excluded to reduce the risk of inaccurate self-reporting. The same BDHQ procedure was applied to both groups.

Eating behavior was evaluated using the eating behavior questionnaire in the Guideline for Obesity (2022) [20]. It consisted of 55-item questions of the following 8 categories: (1) recognition for body weight and constitution, (2) external eating behavior, (3) emotional eating behavior, (4) sense of hunger, (5) eating style, (6) food preference, (7) regularity of eating habits, and (8) their total. All items were rated on a four-point scale ranging from 1 (seldom) to 4 (very often). The scores for these categories were then summed and calculated as total eating behavior [9].

### 2.5. Metabolic Risk Factors

After an overnight fast of ≥12 h, blood was sampled from the antecubital vein and analyzed for triglycerides, total cholesterol, HDL cholesterol, glucose, and insulin in a commercial laboratory (LSI Medience, Tokyo, Japan). We calculated the homeostasis model of assessment-insulin resistance (HOMA-IR), an index of insulin resistance, using the following equation:

HOMA-IR = fasting blood glucose (mg/dL) × fasting insulin (μIU/mL)/405

### 2.6. Statistical Analysis

Since some data showed a non-normal distribution by the Shapiro–Wilk test, all data were shown as medians and 25th to 75th percentiles (Table 1). The Mann–Whitney U test was used to assess the significance of differences between younger and older women. The relationships between IntraMAT content and dietary intake, eating behavior, and other items were analyzed using Spearman’s rank correlation coefficients. The significance of differences was set at <5%. All data were processed using IBM SPSS Statistics 29 (IBM Japan, Tokyo, Japan).

## 3. Results

### 3.1. Characteristics of Participants

The characteristics of the younger and older groups are shown in Table 1. Significant group differences were observed in height, body fat percentage, and IntraMAT content. Regarding dietary intake, energy intake and the percentage of energy from protein and animal protein were significantly higher in the older group than in the younger group. The percentage of energy from carbohydrates was significantly higher in the younger group than in the older group. In terms of eating behavior, motivation for eating, sense of hunger, bad eating habits, diet contents, eating pattern, and total eating behavior were significantly higher in the younger group than in the older group. Among other variables, significant group differences were noted in the levels of triglycerides, total cholesterol, and glucose. No significant group differences were detected in the other items tested.

**Table 1:** Characteristics of younger group and older group.

	Younger Group (*n* = 24)			Older Group (*n* = 25)			
Items	Median	25%	75%	Median	25%	75%	*p*
***Body composition***							
Height (cm)	160.1	155.2	162.5	152.7 **	149.5	154.9	<0.001
Body weight (kg)	51.6	44.9	58.4	51.4	45.2	55.3	0.418
Body fat (%)	26.3	22.4	30.9	32.0 *	27.6	35.3	0.015
BMI (kg/m^2^)	20.2	18.7	22.3	21.9	20.0	23.5	0.091
Waist circumference (cm)	70.5	65.9	78.2	82.5 **	76.8	85.5	<0.001
IntraMAT (%)							
Rectus abdominis	12.6	9.4	15.3	31.6 **	19.6	40.2	<0.001
Abdominal oblique	15.5	12.7	17.2	31.2 **	24.9	36.0	<0.001
Erector spinae	13.1	10.9	15.1	28.4 **	21.3	33.7	<0.001
Iliopsoas	10.1	9.3	12.0	15.0 **	13.8	17.2	<0.001
Average	12.5	11.0	15.0	27.7 **	19.6	32.6	<0.001
***Dietary intake***							
Energy intake (kcal)	1493	1337	1774	1755 *	1539	1880	0.048
Protein (%)	13.9	12.9	16.7	17.5 **	16.1	19.6	<0.001
Animal protein (%)	8.2	6.8	9.8	11.0 **	9.4	12.5	<0.001
Plant protein (%)	6.5	5.6	7.1	6.6	5.8	7.7	0.575
Fat (%)	29.5	25.3	33.0	30.9	28.3	35.7	0.263
Animal fat (%)	13.2	11.8	16.1	15.6	13.5	16.8	0.055
Plant fat (%)	15.8	13.5	18.1	15.6	12.9	17.1	0.589
Saturated fatty acids (%)	7.7	6.8	8.8	8.5	7.3	9.3	0.180
Monounsaturated fatty acids (%)	10.9	9.3	12.0	11.0	9.9	12.0	0.704
Polyunsaturated fatty acids (%)	7.2	5.9	8.1	7.6	6.3	8.8	0.263
Carbohydrates (%)	53.8	49.5	59.2	50.2 *	45.1	52.3	0.025
***Eating behavior***							
Recognition for body weight and constitution (%)	56.3	49.0	66.7	50.0	41.7	58.3	0.056
External eating behavior (%)	50.0	41.7	64.6	41.7 *	36.1	47.2	0.016
Emotional eating behavior (%)	43.8	25.0	56.3	37.5	25.0	43.8	0.252
Sense of hunger (%)	45.8	33.3	54.2	33.3 *	29.2	41.7	0.018
Eating style (%)	52.5	33.8	65.0	30.0 **	25.0	45.0	0.007
Food preference (%)	51.8	46.4	57.1	35.7 **	28.6	46.4	<0.001
Eating pattern (%)	59.4	43.8	66.4	34.4 **	31.3	40.6	<0.001
Total eating behavior (%)	49.8	44.0	58.0	37.7 **	33.6	43.6	<0.001
***Metabolic risk factors***							
Triglycerides (mg/dL)	56.0	38.8	74.3	91.0 **	67.0	105.0	0.002
Total cholesterol (mg/dL)	180.5	157.5	203.0	220.0 **	204.0	242.0	<0.001
HDL cholesterol (mg/dL)	67.0	59.8	80.0	68.0	62.0	82.0	0.535
Glucose (mg/dL)	81.5	78.0	87.3	90.0 **	84.0	99.0	0.001
Insulin (µg/dL)	4.4	3.2	5.9	4.3	2.9	5.7	0.944
HOMA-IR	0.9	0.6	1.4	0.9	0.6	1.3	0.562

BMI: body mass index, IntraMAT: intramuscular adipose tissue, HDL: high-density lipoprotein, and HOMA-IR: homeostatic model assessment for insulin resistance. * *p* < 0.05, ** *p* < 0.01 vs. younger group.

### 3.2. Correlations Between Trunk IntraMAT Content and Other Variables

Table 2 shows correlation coefficients between trunk IntraMAT content and other variables. In the younger group, IntraMAT content was significantly related to HDL cholesterol and insulin (r_s_ = −0.411 and 0.415, *p* < 0.05). In the older group, IntraMAT content significantly correlated with the percentage of energy from protein, sense of hunger, and total eating behavior (r_s_ = −0.410 to 0.412, *p* < 0.05). The other variables tested did not significantly correlate with IntraMAT content (r_s_ = −0.363 to 0.389, n.s.).

## 4. Discussion

The objective of the present study was to derive preliminary hypotheses regarding the associations of IntraMAT content with dietary intake and eating behavior in young and older Japanese women. The main results obtained showed that (1) trunk IntraMAT content significantly correlated with HDL cholesterol and insulin in the younger group, and (2) trunk IntraMAT content significantly correlated with the percentage of energy from protein and eating behavior in the older group. These results suggest that trunk IntraMAT content may be correlated with dietary protein intake and eating behavior, particularly, in older Japanese women.

In the present study, a significant correlation was observed between trunk IntraMAT content and the percentage of energy from protein in older women (Table 2). Previous studies reported that dietary protein affected not only skeletal muscle mass, but also IntraMAT content [21]. Akazawa et al. (2023) demonstrated that an increase in protein intake was significantly related to a decrease in IntraMAT content in the thigh in the older group [3]. Higher protein diets reduce body mass, fat mass, and triglycerides [22] and increase fullness in addition to preserving skeletal muscle mass [23]. Madani et al. (2012) suggested that protein intake improved insulin sensitivity and contributed to the suppression of inflammatory cytokines, such as TNF-α [23,24,25]. Insulin resistance and inflammatory cytokines were shown to correlate with IntraMAT content [12,26]. Collectively, these findings indicate that protein intake inhibits inflammatory cytokines, which, in turn, may be associated with the prevention of or a reduction in IntraMAT accumulation. However, because the present study did not assess inflammatory cytokines, this possibility remains speculative. The mechanisms underlying the relationship between protein intake and IntraMAT content currently remain unclear and, thus, warrant future studies.

Eating behavior is one of the important factors for preventing and treating cardiometabolic diseases, such as metabolic syndrome [1] and type 2 diabetes [10]. Eating behavior, including recognition of body weight and composition, substitute eating, sense of hunger, diet contents, and eating pattern, was found to be significantly worse in individuals with a high visceral adipose tissue content than in those with a low content [10]. The present study firstly found significant positive correlations between trunk IntraMAT content and sense of hunger and total eating behavior scores in the older group (Table 2). In previous studies, individuals with high visceral adipose tissue, metabolic syndrome, type 2 diabetes, or hypertension had worse eating behavior than healthy individuals [1,10]. Eating behavior also significantly correlated with adipocytokine secretion and was suggested to contribute to the development of obesity [27]. Moreover, impaired appetite sensations, such as fullness or hunger, may be factors contributing to overeating and obesity [28]. Accordingly, the relationship between IntraMAT content and eating behavior may reflect an exploratory association, but any physiological interpretation should be regarded as speculative in the present study. Furthermore, the observed relationship may reflect broader age-related differences in health, lifestyle, or functional status rather than a direct relationship with IntraMAT content alone. Therefore, the mechanisms underlying the relationship between IntraMAT content and eating behavior need to be examined in more detail in the future.

In the present study, IntraMAT content significantly correlated with protein intake and eating behavior in the older group only (Table 2). Because the present analyses were exploratory in nature, only correlation analyses were performed in the present study. Therefore, it remains unclear whether this relationship differs by age group. Aging is associated with declines in both metabolic function and the efficiency of protein absorption and utilization, as well as an increase in inflammatory cytokine secretion [29]. Fard et al. (2019) suggested that healthy dietary patterns (a diet high in fruits, vegetables, and whole grains) reduced the risk of frailty in the older (≥65 years), but not in middle-aged and older participants (>45 years) [30]. Older individuals are generally recommended to intake more protein than younger individuals in order to effectively increase and/or maintain physical performance, muscle strength, and health lifespan [31]. Taken together, these findings suggest that the effects of dietary intake on IntraMAT content may be more pronounced in older individuals. However, the present study was cross-sectional and exploratory in nature, with limited participants and analytical approaches. Therefore, further longitudinal or interventional studies are needed to confirm whether age-related differences exist in the associations of IntraMAT content with dietary intake and eating behavior.

As discussed above, this is the first study to show that trunk IntraMAT content significantly correlated with metabolic risk factors, such as insulin and HDL cholesterol, in younger women (Table 2). Miljkovic et al. (2013) reported a significant positive correlation between trunk IntraMAT mass and insulin and HOMA-IR, an index of insulin resistance, in older men [12]. Similarly, Therkelsen et al. (2013) showed significant correlations between IntraMAT content and metabolic risk factors, such as triglycerides and HDL cholesterol, as well as insulin resistance in middle-aged men and women [32]. IntraMAT has been suggested to reduce insulin sensitivity in skeletal muscle through the release of adipokines [33] and the peroxidation of muscle lipids, leading to insulin resistance [34]. A similar underlying mechanism may be involved in the relationships between trunk IntraMAT and metabolic risk factors in young women.

There were several limitations that need to be addressed. First, although multiple variables were analyzed, other factors that may influence IntraMAT content, such as physical activity level, unmeasured dietary factors, and individual lifestyle characteristics, were not fully controlled. As a result, residual confounding cannot be excluded. Second, the cross-sectional design limits causal inference, and it remains unclear whether dietary intake or eating behavior directly contributed to increases or decreases in IntraMAT content. Third, because the present analyses were exploratory in nature, false discovery rate correction was not applied, which should be considered a limitation. Furthermore, the participants in the present study were healthy younger and older women, and not men, patients, or middle-aged individuals, and the sample size was relatively small. Therefore, further studies with larger and more diverse populations are needed.

## 5. Conclusions

The present results suggest that in older Japanese women, trunk IntraMAT content may be related to dietary protein intake and daily eating behavior.

## Figures and Tables

**Table 2:** Correlation coefficients between trunk IntraMAT content and other variables.

	Younger Group		Older Group	
Items	r_s_	*p*	r_s_	*p*
***Dietary intake***				
Energy intake (kcal)	0.075	0.728	−0.124	0.555
Protein (%)	0.248	0.243	−0.410 *	0.042
Animal protein (%)	0.228	0.284	−0.219	0.292
Plant protein (%)	0.153	0.475	−0.363	0.074
Fat (%)	0.071	0.741	−0.179	0.391
Animal fat (%)	0.258	0.223	0.020	0.924
Plant fat (%)	−0.205	0.336	−0.134	0.524
Saturated fatty acids (%)	0.042	0.846	−0.037	0.861
Monounsaturated fatty acids (%)	−0.028	0.897	−0.182	0.385
Polyunsaturated fatty acids (%)	0.115	0.593	−0.164	0.434
Carbohydrates (%)	−0.147	0.493	0.205	0.325
***Eating behavior***				
Recognition for body weight and constitution (%)	0.160	0.457	0.125	0.553
External eating behavior (%)	−0.024	0.913	0.204	0.329
Emotional eating behavior (%)	0.002	0.993	0.353	0.084
Sense of hunger (%)	0.222	0.296	0.404 *	0.045
Eating style (%)	0.361	0.083	0.169	0.419
Food preference (%)	−0.270	0.202	0.320	0.119
Eating pattern (%)	−0.128	0.550	0.389	0.055
Total eating behavior (%)	−0.078	0.716	0.412 *	0.040
***Metabolic risk factors***				
Triglycerides (mg/dL)	0.348	0.096	0.261	0.208
Total cholesterol (mg/dL)	0.204	0.340	0.194	0.354
HDL cholesterol (mg/dL)	−0.411 *	0.046	−0.237	0.254
Glucose (mg/dL)	0.166	0.437	0.274	0.185
Insulin (µg/dL)	0.415 *	0.043	0.206	0.324
HOMA-IR	0.380	0.067	0.210	0.314

IntraMAT: intramuscular adipose tissue, HDL: high-density lipoprotein, and HOMA-IR: homeostatic model assessment for insulin resistance. * *p* < 0.05.

## Data Availability

The datasets used and/or analyzed during the current study are available from the corresponding author upon reasonable request due to legal or ethical reasons.

## Abbreviations

BMI	Body mass index
HDL	High-density lipoprotein
HOMA-IR	Homeostasis model of assessment-insulin resistance
IntraMAT	Intramuscular adipose tissue
MR	Magnetic resonance
TNF-α	Tumor necrosis factor-α
